# Supplementary material for: DUSP10 constrains innate IL-33-mediated cytokine production in ST2hi memory-type pathogenic Th2 cells
Source: Nat Commun. 2018 Oct 12;9:4231. doi: 10.1038/s41467-018-06468-8 (PMC6185962; doi:10.1038/s41467-018-06468-8)

Supplementary Information

DUSP10 constrains innate IL-33-mediated cytokine production in ST2^hi^ memory-type pathogenic Th2 cells

Yamamoto and Endo et.al

**Supplementary Figures**

**
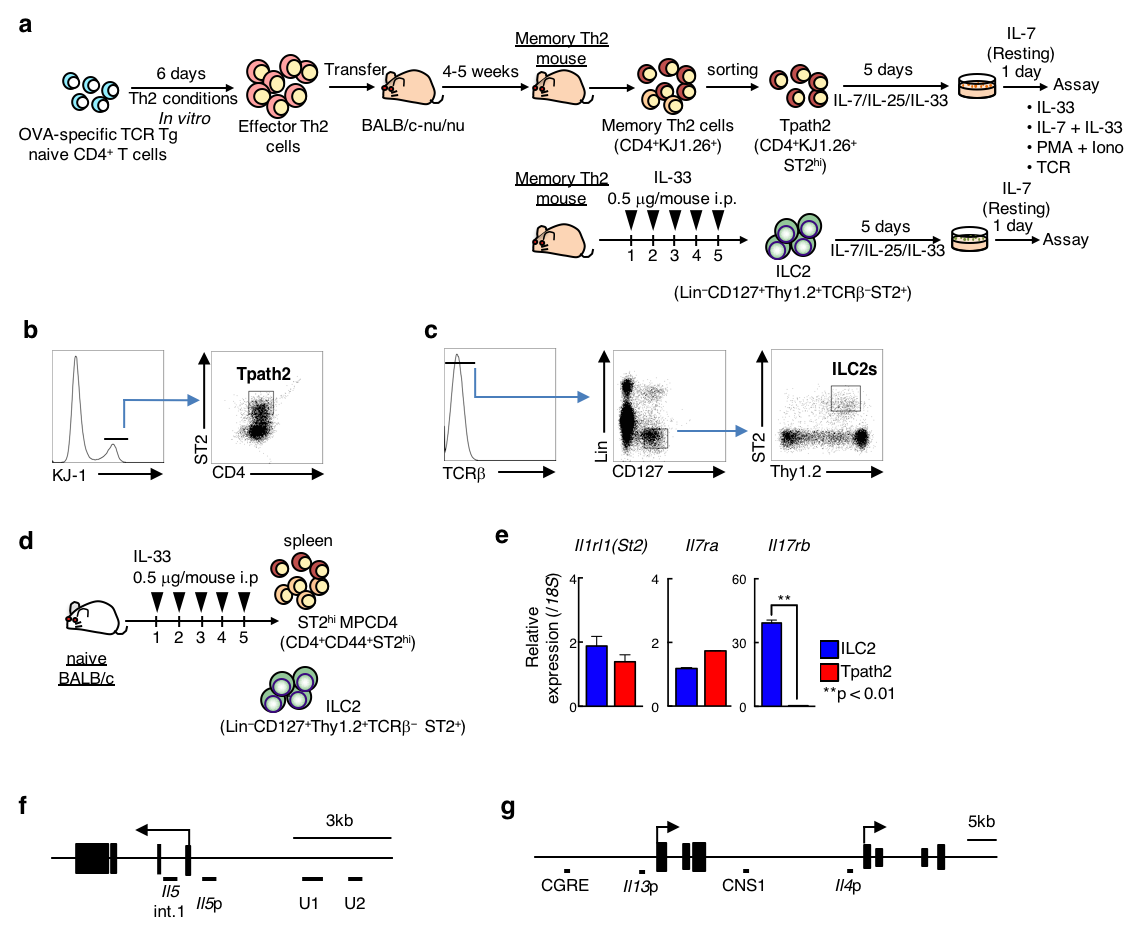
**

**Supplementary Fig. 1. Experimental protocol of ST2^hi^ memory-type pathogenic Th2 cells and ILC2s.**

(**a**) Experimental protocols for the generation of Tpath2 cells and ILC2s. (See also **Cell Preparation** section in the Methods). After isolation, Tpath2 cells and ILC2s were cultured with IL-7, IL-25 and IL-33 for 5 days, and then with IL-7 alone for 24 hours before assays were performed. (**b**) The gating strategy of Tpath2 cells is shown. Splenic cells recovered from memory Th2 mice were stained with anti-CD4-APC, anti-KJ1-FITC and anti-ST2-Bv421 antibodies. CD4^+^KJ1^+^ST2^+^ cells were purified as Tpath2 cells by fluorescence activated cell sorting (FACS). (**c**) The gating strategy of ILC2s cells is shown. After pre-sorting by autoMACS, splenic CD4-negavite cells from IL-33-treated-BALB/c or -memory Th2 mice were stained with anti-TCRβ-PE, anti-Lineage cocktail (containing FITC-conjugated antibodies specific for CD3, Gr-1, CD11b, B220, or Ter119, anti-CD127-APC, anti-Thy1.2-PE/Cy7 and anti-ST2-Bv421 antibodies). Consequently, Lin^-^CD127^+^Thy1.2^+^ST2^+^TCRβ^-^ cells were then isolated as ILC2s by FACS. (**d**) Experimental protocol of generation of ST2^hi^ Memory phenotype CD4^+^ T (ST2^hi^ MPCD4) cells and ILC2s from naïve BALB/c mice. Recombinant mouse IL-33 0.5 μg was injected intraperitoneally to each mouse for consecutive 5 days. Then, ILC2s and ST2^hi^ MPCD4 cells were simultaneously isolated from the spleen. (**e**) Quantitative RT-PCR analysis of surface molecules in ILC2s and Tpath2 cells. Relative expression (normalized to *18S*) is shown with standard deviations. (**f**) Schematic representation of the murine *Il5* gene loci. The locations of primers and probes (upstream region 2 [U2] to *Il5* intron 1 [*Il5* int .1]) and exons are indicated. (**g**) Schematic representation of the murine *CGRE, Il13*p*, CNS1 and Il4*p locus. More than five independent experiments were performed and showed similar results (**e**).


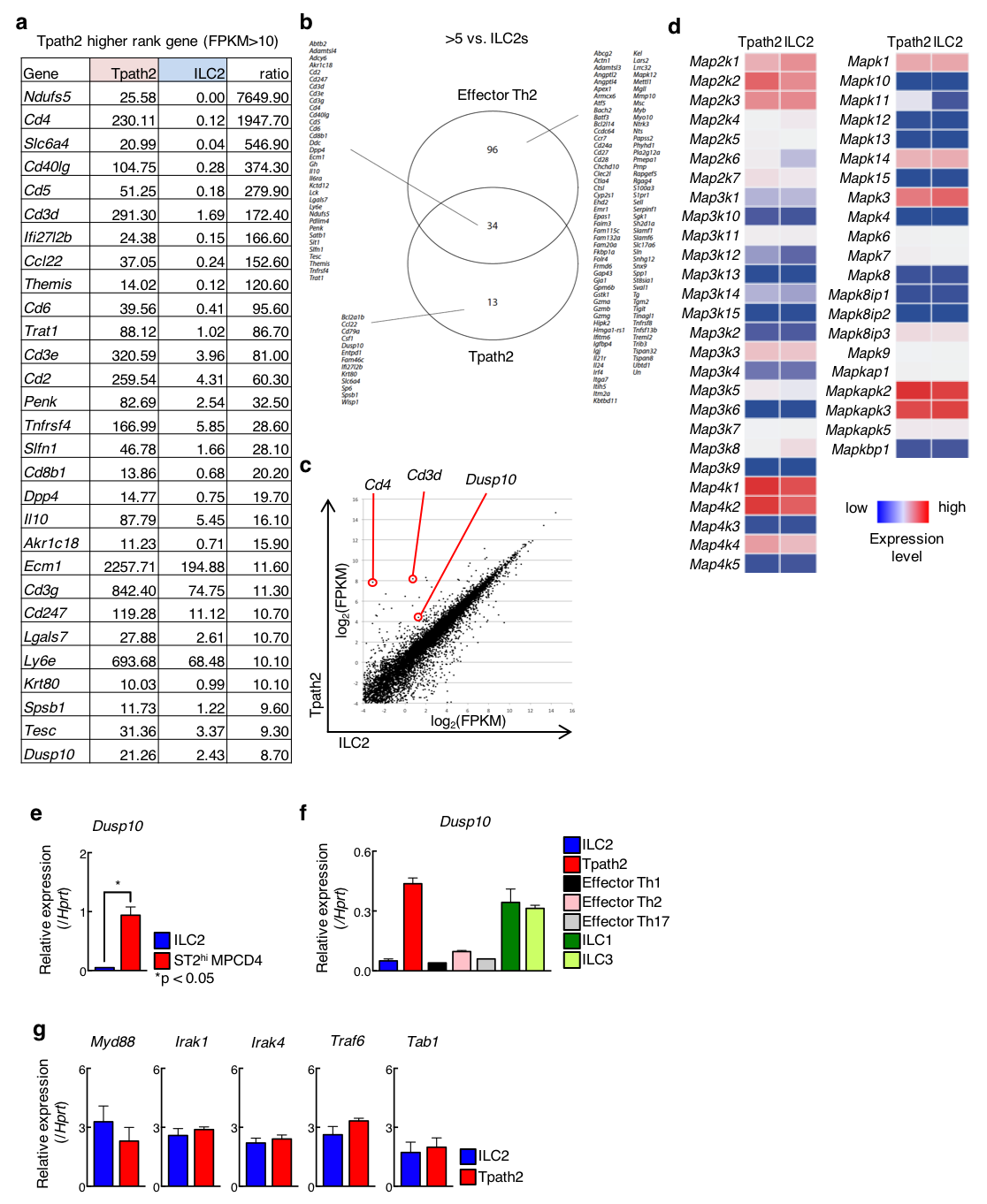


**Supplementary Fig. 2. Selected gene expression profiles from RNA-seq dataset in Tpath2, ILC2s and Effector Th2 cells.**

(**a**) The table shows top ranked genes expressed more highly in Tpath2 cells than ILC2s. Transcripts expressed above 10 FPKM in Tpath2 cells were used. (**b**) Venn diagram shows the overlap of genes in Tpath2 cells and effector Th2 cells at the steady state. Genes expressed more than 5 fold higher in Tpaht2 cells and effector Th2 cells as compared to ILC2s are shown. (**c**) Scatter plot of RNA-seq expression analysis showing a comparison of read counts between Tpath2 cells (Y axis) and ILC2s (X axis) with logarithmic scales. (**d**) A heatmap of the genes encoding members of MAP kinase family in Tpath2 cells and ILC2s. (**e**) Quantitative RT-PCR analysis of *Dusp10* in ST2^hi^ MPCD4 T cells and ILC2s from naïve BALB/c mice. Cell preparation protocol was same as shown in Supplementary Fig. 1d. (**f**) Quantitative RT-PCR analysis of *Dusp10* in ILCs and effector Th cells. (See also Cell Preparation in the Methods). (**g**) Quantitative RT-PCR analysis of indicated genes of intracellular molecules related to IL-33–ST2 signaling pathway in ILC2s and Tpath2 cells. (**e-g**) More than three independent experiments were performed and showed similar results. (*p<0.05). Three technical replicates were included in quantitative RT-PCR analysis.

**
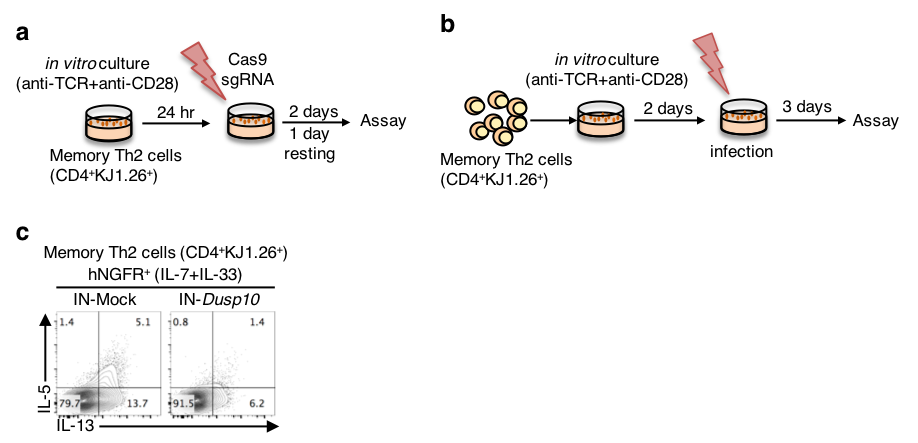
**

**Supplementary Fig. 3. DUSP10 specifically and negatively regulates IL-33–induced IL-5 production in Tpath2 cells.**

(**a**) Experimental protocol of CRISPR/Cas9-mediated gene editing of *Dusp10* in memory Th2 cells. Whole memory Th2 cells were stimulated with anti-CD3 and anti-CD28 and cultured with IL-7, IL-25 and IL-33. After 24 hrs of stimulation, we performed electroporation-based strategy to deliver Cas9/sgRNA. Then, cells were cultured with IL-7, IL-25 and IL-33 for 2 days and after 1day resting with only IL-7, assays were performed. (**b**) Experimental schema of the overexpression of *Dusp10* in memory Th2 cells. Whole memory Th2 cells were stimulated with anti-CD3 and anti-CD28 and cultured with IL-2. On day 2, retroviral vector transfections were performed. Cells were transfected with MOCK and DUSP10. (**c**) Intracellular-staining profiles of IL-5 and IL-13 of hNGFR^+^-infected memory Th2 cells after stimulation with IL-7 plus IL-33 for 6 hrs on day5.

**
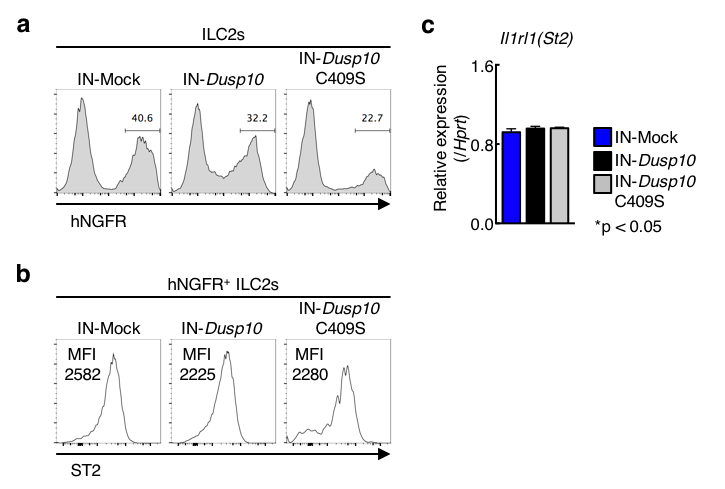
**

**Supplementary Figure 4. *Dusp10* suppresses IL-5 production in ILC2s.**

(**a-c**) Freshly prepared ILC2s from normal BALB/c mice (cell preparation protocol is shown in Supplementary Fig 1c) were infected with a *Mock* (empty vector), *Dusp10*, or C409S mutant-IRES-hNGFR-containing retrovirus and then cultured with IL-7, IL-25 and IL-33. Five days after infection, hNGFR expression was analyzed (**a**). The hNGFR-positive, *Dusp10*-overexpressing ILC2s were enriched by cell sorting. Cell surface ST2 expression (**b**) and mRNA expression of *Il1rl1* (**c**) were analyzed. Three independent experiments were performed with similar results.

**
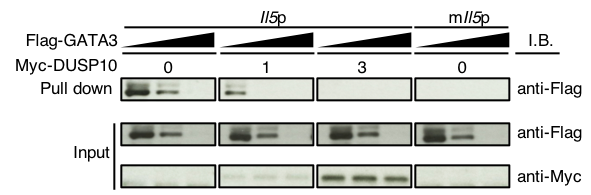
**

**Supplementary Fig. 5. DUSP10 suppresses the DNA binding activity of GATA3.**

293 T cells were transfected with Myc-tagged DUSP10 and Flag-tagged GATA3, and total extracts were subjected to a pull-down assay using WT or mutant *Il5* promoter oligonucleotide as described in the Methods. Immunoblotting of total cell lysates is also shown (Input). Band intensities were measured with a densitometer and arbitrary densitometric units are shown. Two independent experiments were performed with similar results.

**
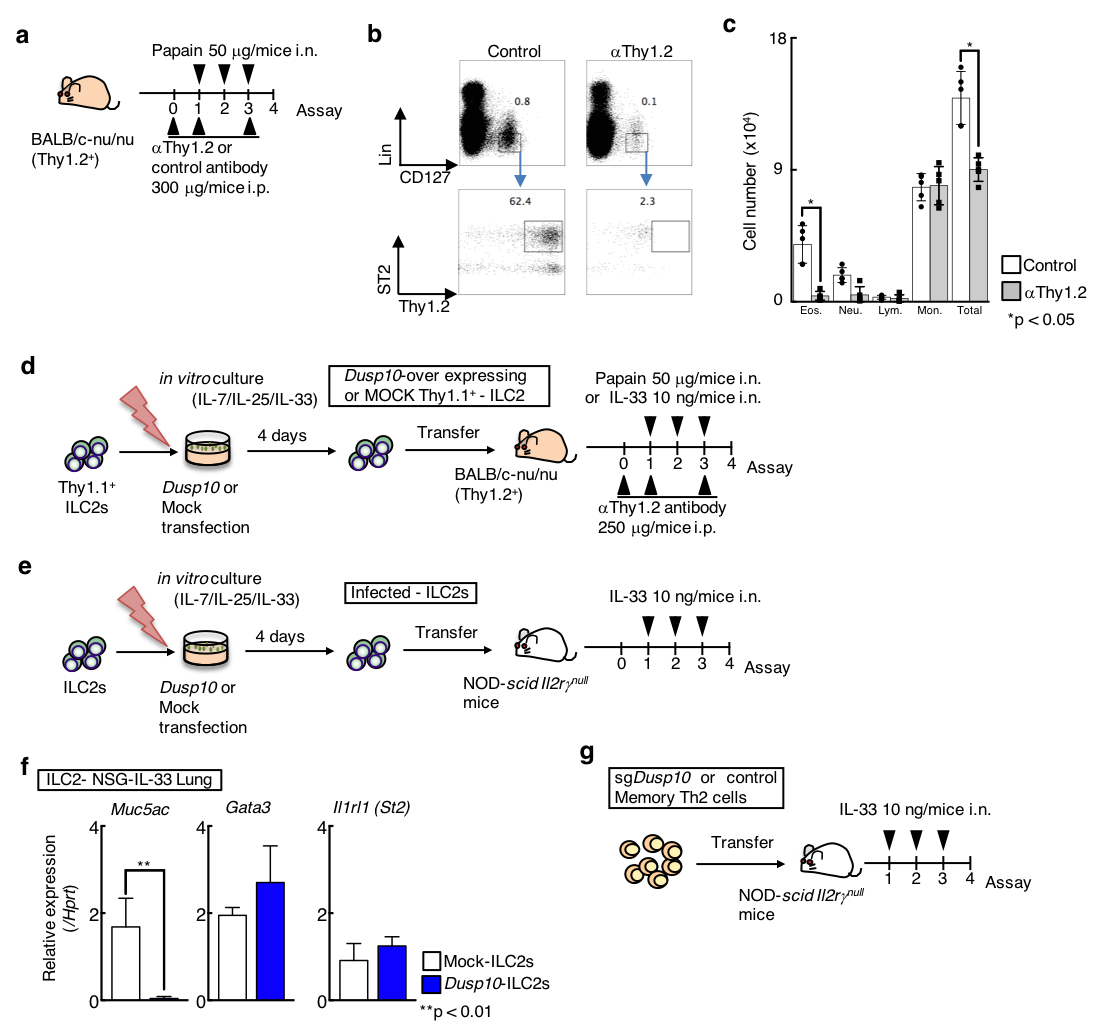
**

**Supplementary Fig. 6. IL-33-induced airway inflammation model.**

(**a**) Experimental protocols for the depletion of intrinsic ILC2 in papain-induced airway inflammation mouse model using Thy1.2 antibody. (**b**) Cell surface expression profiles of Thy1.2 and ST2 on Lin^-^CD127^+^ cells from the lung of isotype control antibody (Control) or anti-Thy1.2 antibody groups (αThy1.2). Two independent experiments were performed and showed similar results. (**c**) The absolute cell number of eosinophils (Eos.), neutrophils (Neu.), lymphocytes (Lym.), and monocytes (Mon.) in the BAL fluid from BALB/c *nu/nu* mice shown in Supplementary Fig. 6a. Mean values (5 mice per group) are shown with standard deviations (*p<0.05). (**d and e**) Experimental protocols for papain and IL-33–induced airway inflammation models (See also ***In vivo* Experiment** section in the Methods). (**f**) Quantitative RT-PCR analysis of *Muc5ac, Gata3* and *Il1rl1* in the lung from Mock-ILC2s and *Dusp10*-ILC2s transferred NSG mice. (**g**) Experimental protocols for IL-33–induced, Tpath2 cell-dependent airway inflammation model. CRISPR/Cas9-mediated *Dusp10* deficient Tpath2 cells were transferred into *NOD-SCID Il2rg^null^* (NSG) mice. These mice received IL-33 intranasally three times (See also ***In vivo* Experiment** section in the Methods).

**Supplementary Fig. 7**

**
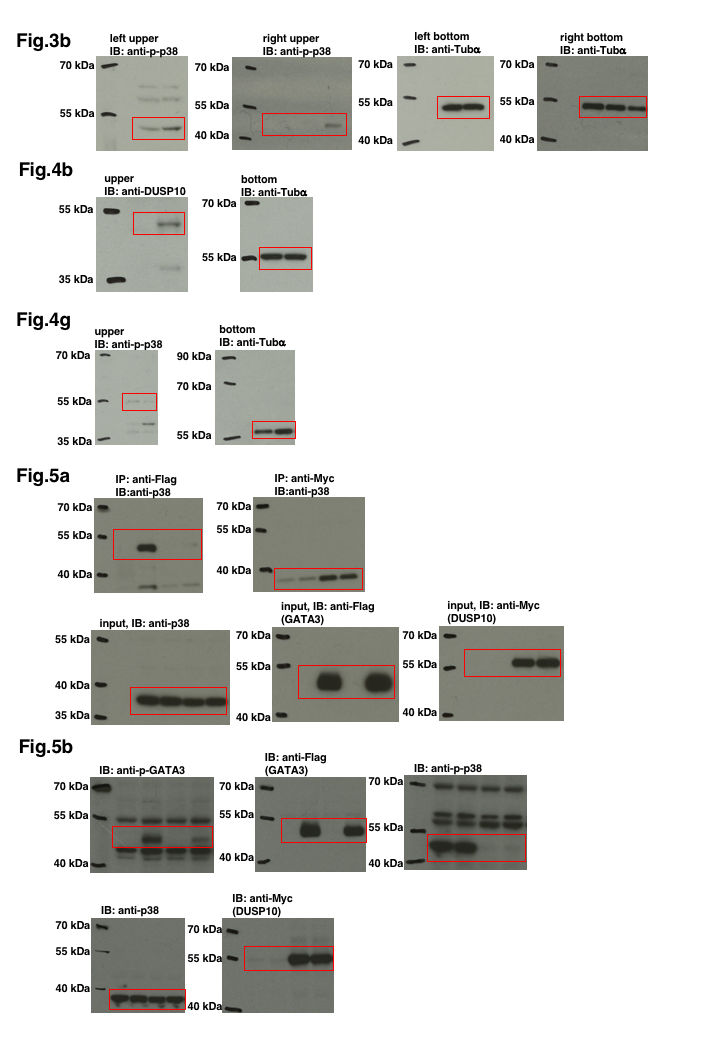
**

**
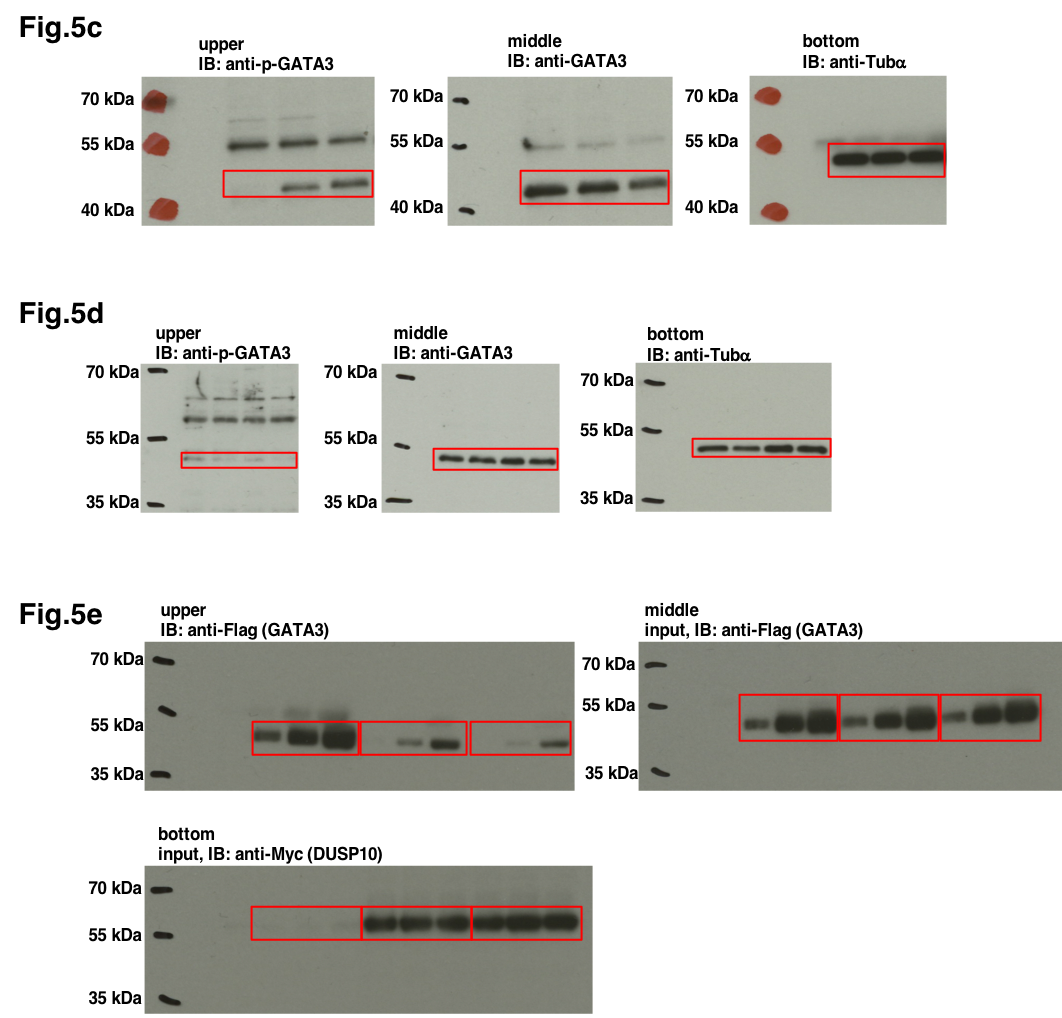
**

**Uncropped scans of western blots are shown with labeled and molecular weight ladder.**

**Supplementary Table 1. Mouse quantitative RT-PCR primer sequences**

Specific primers and Roche Universal Probes used in quantitative reverse transcriptase PCR for mouse genes were as follows.


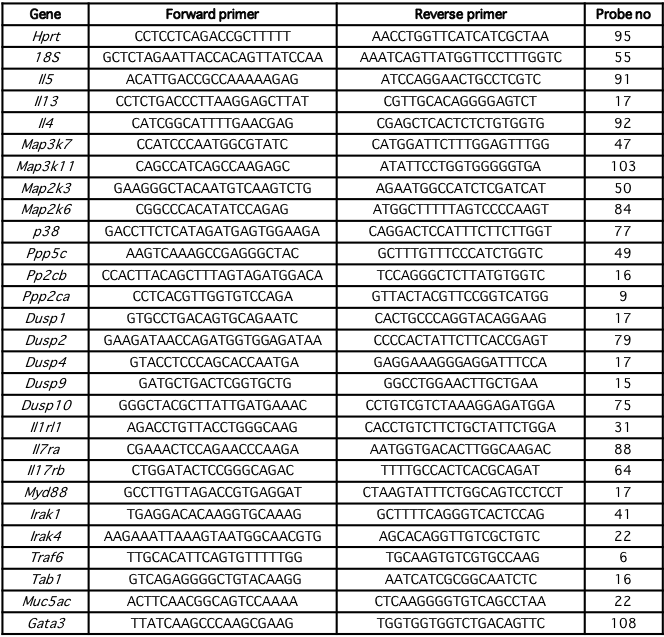


**Supplementary Table 2. ChIP-qPCR primer sequences**

**
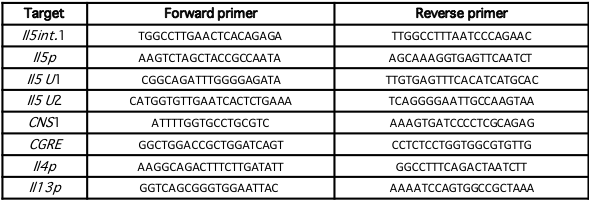
**

**Supplementary Table 3. Primers used for DNA pull-down assay**

GATA3 consensus: 5’ CACTTGATAACAGAAAGTGATAACTCT 3’

Wt *Il5*p: 5’ CCTCTATCTGATTGTTAGCA 3’

Mut*Il5*p: 5′CCTCTATCTGATTAATAGCA 3′

**Supplementary Table 4. List of Commercial antibodies**


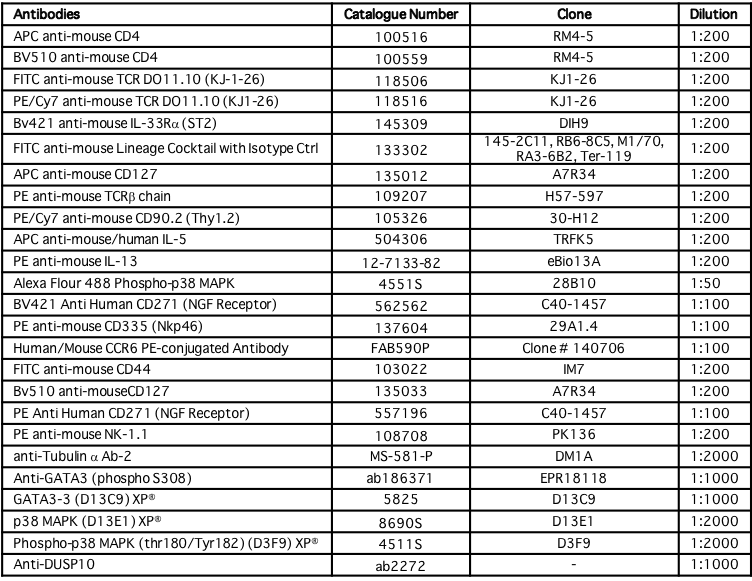

Supplement: Supplementary file 1 — Supplementary Information [file 41467_2018_6468_MOESM1_ESM.docx]
